# Identification of cortical interneuron cell markers in mouse embryos based on machine learning analysis of single-cell transcriptomics

**DOI:** 10.3389/fnins.2022.841145

**Published:** 2022-07-15

**Authors:** Zhandong Li, Deling Wang, Wei Guo, Shiqi Zhang, Lei Chen, Yu-Hang Zhang, Lin Lu, XiaoYong Pan, Tao Huang, Yu-Dong Cai

**Affiliations:** ^1^College of Biological and Food Engineering, Jilin Engineering Normal University, Changchun, China; ^2^State Key Laboratory of Oncology in South China, Department of Radiology, Collaborative Innovation Center for Cancer Medicine, Sun Yat-sen University Cancer Center, Guangzhou, China; ^3^Key Laboratory of Stem Cell Biology, Shanghai Jiao Tong University School of Medicine, Shanghai Institutes for Biological Sciences, Chinese Academy of Sciences, Shanghai, China; ^4^Department of Biostatistics, University of Copenhagen, Copenhagen, Denmark; ^5^College of Information Engineering, Shanghai Maritime University, Shanghai, China; ^6^Channing Division of Network Medicine, Harvard Medical School, Brigham and Women’s Hospital, Boston, MA, United States; ^7^Department of Radiology, Columbia University Irving Medical Center, New York, NY, United States; ^8^Key Laboratory of System Control and Information Processing, Ministry of Education of China, Institute of Image Processing and Pattern Recognition, Shanghai Jiao Tong University, Shanghai, China; ^9^CAS Key Laboratory of Tissue Microenvironment and Tumor, Shanghai Institute of Nutrition and Health, University of Chinese Academy of Sciences, Chinese Academy of Sciences, Shanghai, China; ^10^School of Life Sciences, Shanghai University, Shanghai, China

**Keywords:** cortical interneuron diversity, ganglionic eminences, machine learning, rule learning, embryo

## Abstract

Mammalian cortical interneurons (CINs) could be classified into more than two dozen cell types that possess diverse electrophysiological and molecular characteristics, and participate in various essential biological processes in the human neural system. However, the mechanism to generate diversity in CINs remains controversial. This study aims to predict CIN diversity in mouse embryo by using single-cell transcriptomics and the machine learning methods. Data of 2,669 single-cell transcriptome sequencing results are employed. The 2,669 cells are classified into three categories, caudal ganglionic eminence (CGE) cells, dorsal medial ganglionic eminence (dMGE) cells, and ventral medial ganglionic eminence (vMGE) cells, corresponding to the three regions in the mouse subpallium where the cells are collected. Such transcriptomic profiles were first analyzed by the minimum redundancy and maximum relevance method. A feature list was obtained, which was further fed into the incremental feature selection, incorporating two classification algorithms (random forest and repeated incremental pruning to produce error reduction), to extract key genes and construct powerful classifiers and classification rules. The optimal classifier could achieve an MCC of 0.725, and category-specified prediction accuracies of 0.958, 0.760, and 0.737 for the CGE, dMGE, and vMGE cells, respectively. The related genes and rules may provide helpful information for deepening the understanding of CIN diversity.

## Introduction

Cortical interneurons (CIN) are a group of cells in the human cerebral cortex, which participate in multiple essential biological processes in the human neural system, such as learning (Pi et al., 2013), vision (Pfeffer et al., 2013), and decision making (Wang, 2002). The cerebral cortex of human beings has more than dozen of CIN subgroups, such as multiple kinds of GABAergic interneurons (Kelsom and Lu, 2013). However, for a long time, the biological functions of how CINs are mediated by abundant, diverse cell subgroups has not been well known.

To investigate the complexity of CIN subgroups, the origins of CIN are clearly and reliably revealed, and how they differentiate into different cell subgroups is a premise. Early in 2002, researchers already revealed that most GABAergic interneurons are generated from embryonic forebrain in the cortical subventricular zone and further immigrate to other regions of the brain (Gelman and Marín, 2010). In adult human brains, the CINs originate from three regions, namely, caudal ganglionic eminence (CGE), dorsal medial ganglionic eminence (dMGE), and ventral medial ganglionic eminence (vMGE), with specific cellular components and biological functions (Wonders and Anderson, 2006; Kelsom and Lu, 2013). Although a rough picture about the formation of diverse CINs is emerging, how and when progenitors develop into multiple subgroups of CINs remain controversial. Two major CIN models have been made to interpret the timeline and biological procedures for the generation of CIN diversity (Wonders and Anderson, 2006):

Model 1: The diversity of CIN is invoked in the original region before they migrate.

Model 2: The diversity of CIN is regulated after the migration and by the local environment of each region.

To test and validate these two hypotheses, one effective approach could be to investigate the transcriptomic profiling at single cell level during embryo development (Mi et al., 2018). Recently, Mi et al. (2018) sequenced the transcriptome for cells collected from the three regions of origin of CIN (CGE, dMGE, and vMGE) in the mouse subpallium across two time points (embryonic days 12.5 and 14.5) and then explored the transcriptomic characteristics of the cells.

In this study, as inspired by Mi et al.’s (2018) work, a more sophisticated bioinformatic analysis on the transcriptomic profiling of CIN cells is conducted by using the same data. Our hypothesis is that if cell types (CGE, dMGE, and vMGE cells) in the regions of origin could be predicted by their transcriptomic profiles, then “The diversity of CIN is invoked in the original region” (Model 1); otherwise, “The diversity of CIN is regulated after the migration” (Model 2). Our analysis consists of a series of machine learning algorithms, including the minimum redundancy and maximum relevance (mRMR) (Peng et al., 2005), the incremental feature selection (IFS) approach (Liu and Setiono, 1998), random forest (RF) (Breiman, 2001), and repeated incremental pruning to produce error reduction (RIPPER) (Cohen, 1995). After the analysis, several myoblast-associated genes (e.g., Lhx8, Calm1, Hmgb1, Meis2, Nr2f2, Basp1, BTAC, ZIC1, NR2F1, and RPS29) were identified and well interpreted via existing literature, and 23 quantitative rules were established to quantify the distribution of identified genes within the regions of origin.

## Materials and methods

### Study design

**FIGURE 1:**
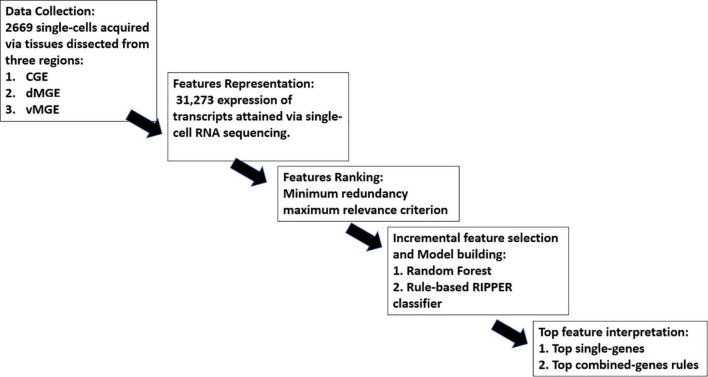
Overall workflow of the study.

Our study consisted of five parts, namely, (1) data collection, (2) feature representation, (3) feature ranking, (4) IFS and model building, and (5) top feature interpretation, as shown in Figure 1. More details are presented in the following sections.

### Dataset

The dataset consisted of 2,669 single cells downloaded from Gene Expression Omnibus (GEO) under the accession number of GSE109796 (Mi et al., 2018), which contained RNA sequencing reports of 2,669 single cells acquired from tissues dissected from the subpallium of mouse embryos. The 2,669 single cells were divided into three cell types according to their regions of origin in the subpallium, namely, CGE cells, dMGE cells, and vMGE cells, whose numbers were 856, 957, and 856, respectively.

### Feature representation

In the downloaded data, RNA sequencing for each cell was recorded by 37,310 reads per kilobase per million mapped reads (RPKM) values. The RPKM values of 6,037 transcripts showed zeros in all cells; thus, they were removed. Finally, each sample (each cell) was represented as a numeric vector of 31,273 RPKM values (37,310-6,037 = 31,273) and used as input in the following computational analysis.

### Feature ranking

As mentioned in section “Feature representation,” lots of features were used to represent each cell sample. Clearly, not all features were related to the classification of cell types. A feature analysis procedure was necessary. In our study, the mRMR method (Peng et al., 2005) was employed for completing this task. mRMR is a filter-based feature analysis algorithm that could retain feature relevance to classification labels while reducing redundance among features, that is, maximizing the correlation between features and labels while minimizing the correlation between features themselves. However, such purpose is difficult to achieve because the problem is NP-hard. Thus, mRMR gives a heuristic scheme, which sorts feature in a list, named mRMR feature list. At the beginning, this list is empty. The mRMR method selects one feature, which has highest relevance to classification labels and lowest redundance to already-selected features, and appends such feature to the list in each round. This procedure stops until all features are in the list. Evidently, features with the top ranks in the list had the most relevance to classification labels and less correlation to the rest of the other features. Thus, they can comprise a compact feature subspace to represent samples.

This study adopted the mRMR program reported in http://home.penglab.com/proj/mRMR/. It was performed with its default parameters.

### Feature selection and model building

As mRMR method only sorts features in the mRMR list. Which features are important and can be picked up for constructing classifiers is still a problem. In view of this, the IFS method (Liu and Setiono, 1998) followed to analyze this list in this study. It is a feature screening approach to determine the optimal number of features. To perform IFS on the mRMR feature list, a series of feature subsets with a step of 10 features was first generated, that is, the *i*-th feature subset contained the first 10 × *i* features from the mRMR list. For each subset, a classifier was trained based on one classification algorithm and samples consisting of the features from this feature subset. This classifier was further evaluated by 10-fold cross-validation (Kohavi, 1995; Chen et al., 2017; Zhou et al., 2020; Zhu et al., 2021). Then, the classifier giving the optimal performance can be obtained. This classifier was termed as the optimal classifier, whereas the features used in this classifier were called optimal features.

As mentioned above, a classification algorithm is necessary for IFS method. Here, two widely used machine-learning classification algorithms were adopted for building classifiers in this study, namely, the RF (Breiman, 2001) and RIPPER (Cohen, 1995) algorithms. RF is a meta classifier that contains many decision trees, and each tree is grown from a bootstrap set with a randomly selected feature subset. For making a prediction, its output label is determined by aggregating votes from different decision trees. Several differences always exist between different decision trees in the forest. RF usually averages the prediction results of all decision trees to reduce errors and avoid overfitting. Although this slightly increases the bias and loses some interpretability, it improves prediction performance. RF is always quite efficient to construct classifiers and has wide applications in the fields of bioinformatics (Pan et al., 2010; Jia et al., 2020; Liang et al., 2020; Chen et al., 2021; Zhang et al., 2021c). In this study, we directly employed the tool “RandomForest” in Weka (Frank et al., 2004), which implements the RF. For convenience, default parameters were used, where the number of decision trees is set to 100.

Although the RF classifier can be efficient to classify CIN cells, it cannot give useful clues to uncover differences of CIN cells from different regions. In view of this, we further employed another classification algorithm, RIPPER. In fact, it is a rule-based algorithm that could generate classification rules to predict cells into different region-based groups. In RIPPER, the training set is first separated into the growing set and pruning set. Then, the rule grows, and the prune phase is repeated until no positive samples are left in the growing set. During the process, one rule is generated by greedily adding conditions to the rule. It predicts the classification of new data based on the interpretable classification IF–ELSE rules. Although the RIPPER classifier is generally weaker than RF classifier, it has its own merits. For CIN cells investigated in this study, the rules contained in the RIPPER classifier can clearly indicate the patterns of cells in different regions, giving new insights to study CIN cells. Thus, RIPPER has also been applied to analyze some complicated biological or medical systems (Li et al., 2020; Zhang et al., 2020, 2021a). Likewise, the tool “JRip” in Weka (Frank et al., 2004) was directly used in this study, which implements the above RIPPER. Also, it was executed using default parameters.

### Model interpretation

In our study, the interpretation of model consisted of two parts, interpretation on (1) single-gene and (2) combined-gene rules. Single-gene interpretation focused on the optimal genes (i.e., the feature in the sample vector) selected by IFS with RF, whereas interpretation on combined-gene rule focuses on the optimal rules outputted by IFS with RIPPER. Our interpretation was based on comprehensive literature reviewing on previous works.

### Performance measurement

Two quantitative metrics were used to indicate the performance of different models, namely, prediction accuracy (ACC) and Matthew correlation coefficients (MCC) (Matthews, 1975; Gorodkin, 2004; Zhao et al., 2018; Pan et al., 2021; Zhang et al., 2021b). ACC could directly show the proportion of correctly predicted samples among all samples. However, this measurement is not very correct if the sizes of classes are of great differences. In this case, MCC is more objective. To calculate such measurement, two matrices, say *X* and *Y*, should be constructed first, where *X* denotes the true class of each sample and *Y* represented the predicted class of each sample. Accordingly, MCC can be computed by


(1) $$M\,C\,C=\,\frac{c\,o\,v\,\left(X,Y\right)}{\sqrt{c\,o\,v\,\left(X,X\right)\,c\,o\,v\,\left(Y,Y\right)}},$$

where cov(●) stands for the correlation coefficient of two matrices. The return values of ACC and MCC are continuous values with range of 0–1 and -1 to 1, respectively. The higher the ACC and MCC values are, the better performance for the classifiers.

In addition, we also employed the individual accuracy to measure the performance of each classifier. For each class, the individual accuracy of this class is defined as the proportion of correctly predicted samples in this class among all samples in this class. Evidently, high individual accuracy indicates the good performance of one classifier on some class.

## Results

In our study, 2,669 single-cell samples were collected, and each cell was represented as a numeric vector with 31,273 features. Each feature in the sample vector corresponded to the expression of one gene. The 2,669 samples were divided into three categories, CGE cells (856, 32%), dMGE cells (957, 36%), and vMGE cells (856, 32%). The entire analysis procedures are illustrated in Figure 1.

### Results of feature ranking

The dataset containing 31,273 features was first analyzed by the mRMR method. The mRMR feature list was obtained, which is provided in Supplementary Table 1. Features with high ranks were essential for distinguishing CIN cells.

### Prediction performance

Based on the mRMR feature list, RF and RIPPER were employed to build the classifiers in the approach of IFS.

**FIGURE 2:**
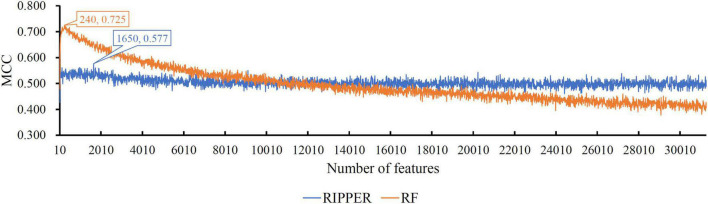
The IFS curves of RIPPER and RF. RIPPER can reach the highest MCC of 0.577, whereas RF can reach the highest MCC of 0.725.

**FIGURE 3:**
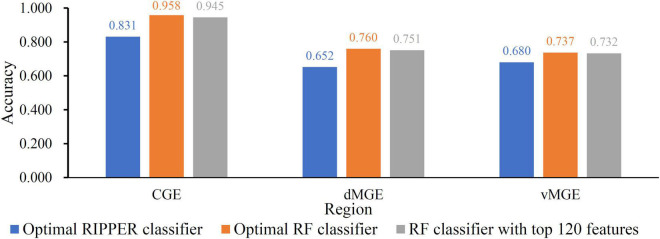
The Individual accuracies of some key classifier. Two RF classifiers provide almost equal performance and are superior to the RIPPER classifier.

**FIGURE 4:**
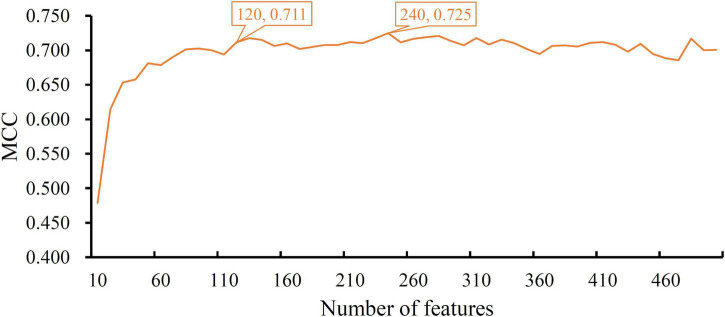
The IFS curve of RF between 10 and 500. The RF classifier with top 120 features yielded a little lower MCC than the classifier with top 240 features, which is the optimal RF classifier.

**FIGURE 5:**
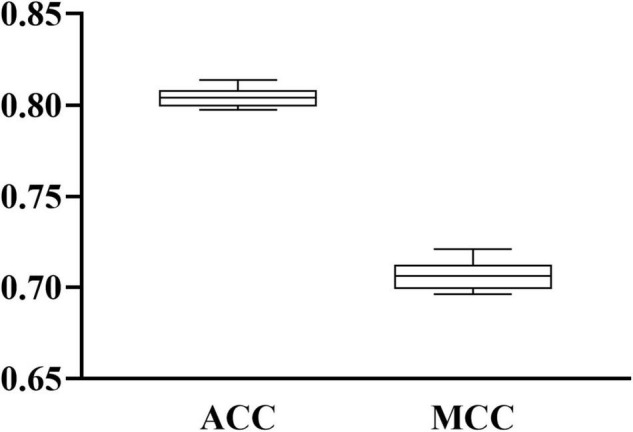
Box plot to show the performance of RF classifier with top 120 features under 10-fold cross-validation for 10 times. ACC and MCC vary in a small range, indicating the stability of the classifier.

When RF was selected in the IFS method, we constructed several RF classifiers on different feature subsets. The performance of these classifiers is listed in Supplementary Table 2. For an easy observation, an IFS curve was plotted, as shown in Figure 2, where *x*-axis stands for the number of features in the feature subset and *y*-axis stands for the MCC. The highest MCC was 0.725 when top 240 features were used. Accordingly, these 240 features were the optimal features for RF and an optimal RF classifier was constructed with these optimal features. The ACC of this classifier was 0.816, as listed in Table 1. The individual accuracies on CIN cells in three regions are shown in Figure 3. Most CGE cells were correctly predicted with individual accuracy higher than 0.950, whereas the other two individual accuracies were not very high. As a whole, this classifier gave good performance. However, this RF classifier used lots of features. It would not be efficient if lots of samples were inputted. In view of this, we enlarged the IFS curve between 10 and 500, as shown in Figure 4. It can be observed that the curve followed a sharp increasing trend when the number of features were small. After that, the curve became stable. When top 120 features were adopted, the MCC reached 0.711, which was a little lower than that yielded by the optimal RF classifier (0.725). As for ACC, it was 0.807 (Table 1), also slightly lower than that produced by the optimal RF classifier (0.816). The individual accuracies of this classifier are illustrated in Figure 3. All were a little lower than those of the optimal RF classifier. These results indicated that the RF classifier with top 120 features provided almost equal performance to the optimal RF classifier. However, it involved much less features, indicating higher efficiency. This RF classifier can be an efficient tool to classify CIN cells. Furthermore, the top 120 features were much more important than the following 120 features. It was valuable to investigate their relationships to CIN cells. Furthermore, to further assess such latent tool, it was tested by additional 10-fold cross-validation for ten times. A box plot, as shown in Figure 5, was drawn for obtained ACC and MCC values. Evidently, they were changed in a small range, indicating the stability of the tool.

**TABLE 1:** Performance of some key classifiers.

Classification algorithm	Number of features	ACC	MCC
RIPPER	1,650	0.718	0.577
RF	240	0.816	0.725
RF	120	0.807	0.711

Although the above RF classifiers provided satisfied performance, they provided less even no useful clues to uncover differences of CIN cells in different regions because RF is a black-box algorithm. Thus, we also adopted RIPPER in the IFS method. The performance of RIPPER classifiers on all tested feature subsets is provided in Supplementary Table 2. An IFS curve was plotted in Figure 2 to show their performance. The highest MCC was 0.577 when top 1,650 features were used. Thus, these features were the optimal features for RIPPER and the optimal RIPPER classifier was built based on them. The ACC of this classifier was 0.718, as listed in Table 1. Evidently, MCC and ACC were much lower than those of above RF classifiers. As for the individual accuracies, they are illustrated in Figure 3. They were also much lower than those of the above RF classifiers. All these indicated that the optimal RIPPER classifier was much inferior to above two RF classifiers. However, some useful information can be extracted from this RIPPER classifier, which would be listed in section “Top feature interpretation.” This information was helpful to uncover the difference of CINs cells in different regions.

### Top feature interpretation

With the help of IFS and RF, we extracted 120 essential features, which are the first 120 features listed in Supplementary Table 2. These features were deemed to play essential roles to distinguish CIN cells in different regions. Their corresponding genes can be essential single-genes to classify CIN cells.

Furthermore, in our study, RIPPER was employed to generate classification rules to reveal how features interacted with one another, thus predicting cell types. The optimal RIPPER classifier used top 1,650 features. Cell samples represented by these features were learnt by RIPPER, resulting in 23 rules, as listed in Table 2. 12 rules were for CGE cells, 9 rules were for vMGE cells and the last rule was for dMGE cells. CGE cell rules ranked higher than the two MGE cell rules, which complied with the observation that CGE cells are easier to be distinguished by their transcriptomic characteristics.

**TABLE 2:** The 22 classification rules generated by RIPPER for predicting cell types.

Index	Rule	Label
1	NKX2-1 ≤ 3.6524) and (MT-TM ≥ 1.4699) and (MEIS2 ≥ 0.7558) and (H3F3B ≥ 2019.223223)	Caudal ganglionic eminence
2	(FOXP2 ≥ 0.0047) and (NKX2-1 ≤ 5.5415) and (TMSB10 ≤ 76.7268) and (NR2F1 ≥ 25.2511)	Caudal ganglionic eminence
3	(NKX2-1 ≤ 12.9594) and (RPS20 ≤ 17.1104) and (SLC7A11 ≥ 0.0538) and (PID1 ≥ 0.4191) and (LHX8 ≤ 0.0839)	Caudal ganglionic eminence
4	(NR2F2 ≥ 0.2374) and (LHX6 ≤ 0.3856) and (5730494M16RIK > = 14.4383) and (LHX8 ≤ 7.0592)	Caudal ganglionic eminence
5	(FOXP2 ≥ 0.1294) and (EPHA5 ≥ 25.8453) and (RPS9 ≤ 159.5439) and (DCX ≥ 49.7284)	Caudal ganglionic eminence
6	(NR2F2 ≥ 2.2717) and (EPHA5 ≥ 3.4019) and (LHX8 ≤ 0) and (MEIS2 ≥ 18.9392) and (NCAPH ≤ 2.4094)	Caudal ganglionic eminence
7	(ENC1 ≤ 1.8862) and (NKX2-1 ≤ 12.0905) and (STX7 ≥ 0.3228) and (CALM1 ≤ 546.2493) and (SOX6 ≤ 2.8190)	Caudal ganglionic eminence
8	(GM6180 ≥ 36.7994) and (FOXP2 ≥ 3.5745) and (GM13340 ≥ 34.469118)	Caudal ganglionic eminence
9	(EPHA5 ≥ 0.1196) and (LHX8 ≤ 0.1496) and (GM15266 ≤ 19.2334) and (GM1821 ≤ 89.839694) and (CALM1 ≤ 744.1502)	Caudal ganglionic eminence
10	(BIRC6 ≤ 0) and (NKX2-1 ≤ 10.7393) and (CRIP2 ≥ 400.8077) and (ZFP238 ≥ 6.1810) and (GM10039 ≥ 12.3788)	Caudal ganglionic eminence
11	(ARRDC3 ≥ 368.6154) and (PTPRS ≤ 24.5126) and (ZFP238 ≤ 70.9705)	Caudal ganglionic eminence
12	(ZFP503 ≥ 0.1111) and (MAP4K4 ≤ 7.0016)	Caudal ganglionic eminence
13	(LHX8 ≥ 1.4487) and (ZIC1 ≥ 0.0111)	Ventral medial ganglionic eminence
14	(LHX8 ≥ 0.3736) and (NR2F1 ≤ 9.4921) and (ZSWIM5 ≤ 0.0334)	Ventral medial ganglionic eminence
15	(NR2F1 ≤ 15.6811) and (CD24A ≥ 2.7069) and (BASP1 ≤ 66.0865) and (PKIA ≤ 1.8492)	Ventral medial ganglionic eminence
16	(PAK3 ≤ 0.7899) and (RPS18 ≥ 4.9001) and (PID1 ≥ 1.2578)	Ventral medial ganglionic eminence
17	(YWHAZ ≥ 1.8915) and (SEPT11 ≤ 7.8189) and (GM13604 ≤ 0.2081) and (H2AFV ≥ 1305.9678)	Ventral medial ganglionic eminence
18	(PAK3 ≤ 0.0474) and (CFL1 ≥ 31.1042) and (GTF2A1 ≤ 20.8911) and (LHX6 ≤ 5.6025)	Ventral medial ganglionic eminence
19	(GM15266 ≤ 63.0475) and (MT-RNR2 ≥ 9387.3828)	Ventral medial ganglionic eminence
20	(CITED2 ≤ 35.2427) and (GM10718 ≤ 1.0898) and (2610017I09RIK ≤ 2442.7505) and (GSK3B ≤ 1.5164)	Ventral medial ganglionic eminence
21	(UBE2QL1 ≥ 1.5601) and (3110003A17RIK ≥ 20.2789) and (RTN1 ≤ 4.7837)	Ventral medial ganglionic eminence
22	(GM3511 ≥ 22.9860) and (PTMA ≥ 2.2359) and (LHX6 ≤ 47.9178)	Ventral medial ganglionic eminence
23	Others	Dorsal medial ganglionic eminence

## Discussion

As mentioned above, 120 features for the RF classifier for CIN cell types were filtered out. The transcriptomic level profiling of candidate genes was associated with CIN development. The literature was searched for the top 30 features, and these features were supported by previous studies as potential biomarkers. Considering length restriction, 10 features were selected for detailed discussion, as listed in Table 3. A series of quantitative rules was also identified for detailed prediction on CGE, dMGE, and vMGE cells, laying a foundation for further analyses in this field.

**TABLE 3:** Details of essential genes.

Gene symbol	Description	Rank in the feature list
Lhx8	LIM homeobox 8	2
Calm1	Calmodulin 1	4
Hmgb1	High mobility group box 1	9
Meis2	Meis homeobox 2	12
Nr2f2	Nuclear receptor subfamily 2 group F member 2	22
Basp1	Brain abundant membrane attached signal protein 1	7
Actb	Actin beta	23
Zic1	Zic family member 1	30
Nr2f1	Nuclear receptor subfamily 2 group F member 1	15
Rps29	Ribosomal protein S29	18

### Genes associated with cortical interneurons

**Lhx8**, the top predicted candidate, is a member of LIM-class homeobox genes. According to recent publications, Lhx8 contributes to the regulation of neuronal Shh expression in MGE. Such gene has also been identified to prevent Nkx2-1 expression in a subset of pallial interneurons (Flandin et al., 2011), suggesting that Lhx8 plays an important role in regulating the early born MGE neurons. In addition, Lhx8 has a special function in the development of neurons. Lhx8 is a major source of cholinergic neurons, and Zhao et al. (2003) proved that Lhx8 is quite essential during cholinergic neurons’ development in the forebrain. Integrating the literature supports mentioned above, speculating that Lhx8 as one of our candidates has differential profiling patterns in MGE neuron cells and other cells, consistent with our prediction results, is quite reasonable.

**Calm1**, another identified CIN-associated gene encodes a kind of calmodulin (CaM), an effective calcium ion sensor and signal transductor. Neuronal migration is associated with our predicted gene, which may further indicate the potential contribution of our predicted genes on the development of the nervous system. Kobayashi et al. (2014) reported that Calm1 acts as a specific regulator in the tangential and radial migration of mouse neuron cells. Neuronal migration is a key process in the developing and adult brain, and Khodosevich et al. (2009) indicated that the knockdown of Calm1 affects neuroblast migration to the OB, suggesting that Calm1 can be an important gene in the regulation of neuron development. Integrating the literature supports mentioned above, Calm1 is confirmed to be a CIN-associated gene.

Another identified gene is **Hmgb1**, which is like histones among the most important chromatin proteins. Hmgb1, a novel cytokine-like mediator, is associated with microglial activation (Kim et al., 2006). According to recent publications (Kim et al., 2011), Hmgb1 participates in the regulation in danger signaling and cell death control, acting as a ubiquitously expressed non-histone DNA-binding protein. In addition, Hmgb1 plays various roles in different stages of brain development (Ping et al., 2012). During early brain development, fore brain development including neurite outgrowth is also associated with Hmgb1. During aging, it is associated with injury induced inflammation. Integrating the literature supports mentioned above, Hmgb1 is suggested to be an important feature to classify the different types of CIN, including CGE, dMGE, vMGE. Overall, effective gene Hmgb1 may also have the capacity to distinguish CGE, dMGE, and vMGE cases.

**Meis2**, the next predicted gene, is associated with the development of neural crest cells (Machon et al., 2015). In early brain development processes, Meis2 participates in the positive and negative regulation of feed-back and feed-forward loops (Agoston et al., 2012). However, Meis can interact with various trophic factor signaling pathways during neuron differentiation (Bouilloux et al., 2015), suggesting that Meis can be an essential factor in development of neuron cells. Therefore, our predicted gene, Meis, may be identified as a special factor in classifying CGE, dMGE, and vMGE cases.

**Nr2f2** is the following predicted gene in our predicted list. Nr2f2, also known as COUP transcription factor 2, participates in development of CIN subtypes (MGE). Hu et al. (2017) indicated that the expression level of Nr2f2 is associated with time-dependent specification of layer STT and the survival of neuroepithelium during brain development. Fuentealba et al. (2010) also proved that as a transcription factor, such gene contributes to the cell type specific development and differentiation control, especially for neurons. Integrating the literature supports mentioned above, speculating that Nr2f2 as a predicted gene may be differentially expressed in CGE, dMGE, and vMGE cases is quite reasonable, validating our prediction.

Another screened out biomarker in our optimal candidates is **Basp1**, also known as GAP-23, which is a well-known member from the family of growth-associated proteins and associated with the protein GAP-43 that is shown to regulate neural cell adhesion molecule controlled outgrowth of neurite. In addition to shared biological functions with GAP-43, Basp1 contributes to the regulation of cellular morphology for plasma membrane (Korshunova et al., 2010). In addition, membrane-binding Basp1 oligomers are associated with the physiological or pathological in-ion channel activity (Ostroumova et al., 2011). Integrating the literature supports mentioned above, Basp1 is also associated with the development of neuron cells. Overall, Basp1 as another predicted gene can also distinguish such three regions in CINs.

Beta-actin (**Actb**) is the next gene related to classify the neuron cells. Beta-actin is a conserved protein of six different actin isoforms regulating cell motility, structure, and integrity. Elongating growth is regulated by our predicted gene Actb via axon branching and translation (Donnelly et al., 2013). Beta-actin can affect the development of neuron cell via essential biological processes for brain development. Beta-actin is a kind of microtubule motor protein during dendritic transport. Integrating the reports mentioned above, beta-actin contributes to the development of neuron cells, suggesting that our predicted gene, BTAC, may be identified as a special factor in distinguishing different CIN regions.

**Zic1** is a member of the zinc finger of the cerebellum Zic protein family. It is classified as a Zic protein due to the conservation of the five C2H2 zinc fingers, and the correct function of this protein is critical for early development. Dipietrantonio and Dymecki (2009) proved that Zic1 levels in pontine gray neurons play an important in the development of pontocerebellar circuit. Therefore, Zic1 can influence the development of brain neurons by pontocerebellar circuity. Zic1 can activate and regulate neural crest development and differentiation at transcriptomic level (Milet et al., 2013; Bae et al., 2014). ZIC1 may be identified as a special factor in distinguishing CGE, dMGE, and vMGE cases.

The next two predicted genes, **Nr2f1** and **Rps29**, are associated with optic atrophy syndrome (Henning et al., 2016) and neuronal gene orthopedic protein (Taylor et al., 2012). Optic atrophy syndrome is a kind of neuronal disease, and orthopedic protein is a protein related to the development of neuron. Overall, these two genes participate in neuron development. Therefore, they can be the factors in classifying CGE, dMGE, and vMGE cases.

### Rules associated with cortical interneurons

In addition to such feature analysis, 22 quantitative rules for brain region clustering for CGE, dMGE, and vMGE cells were identified. Some expression tendencies were confirmed and validated by recent publications. For further validation on such quantitative rules and parameters, the GEO database was screened for accurate FPKM screening. The detailed discussion of each quantitative rule is shown below.

The top 11 rules are associated with the identification of CGE cells, involving **Nkx2-1**, **Foxp2**, **Lhx8**, and **Epha5**. A relatively high expression level of FOXP2 (FPKM > 0) and EPHA5 (FPKM > 3.4), and low expressions of NKX2-1 (FPKM < 3.65) and LHX8 (FPKM < 0) may indicate such CIN case turns out to be a CGE case. According to the biological functions of such genes, FOXP2 is associated with multiple essential functions in human beings including speech and language phenotypes (Rodenas-Cuadrado et al., 2018). Epha5 is an effective kinase from the Eph family, which plays important roles in neural development (Das et al., 2016). Therefore, high expression of these genes can promote the neural development. Our predicted transcription factor NKX2-1 plays a special role in the specification of subsets of cortical, stratal, and pallidal neurons (Magno et al., 2017). Therefore, detected expression of these genes is regarded an effective quantitative filter for the identification of CGE cells.

The following rules contribute to the identification of vMGE cells. Parameters **Lhx8** and **Pak3** are abnormally expressed as potential biomarkers of such group of cells. As reported, a relatively higher expression level of Lhx8 (FPKM > 1.45) and low expression level of PAK3 (FPKM < 0.79) may indicate such CIN case turns out to be a vMGE cells.

According to the biological functions of such genes, a feedback loop between Lhx8 and NGF is associated with the cholinergic functions and may participate in learning and memory (Tomioka et al., 2014). Moreover, PAK3 as a GTPase regulated enzyme has alternative kinase activity with different splicing variants (Combeau et al., 2012), indicating that PAK3 has a critical function in the development of neuron cells. Therefore, detected expression of these genes is regarded a potential parameter for the distinction of CINs.

For cells not following the expression patterns discussed above, they were predicted as dMGE cells.

### Comparison with previously reported cortical interneuron associated biomarkers

Previously, two studies about cortical interneuron associated biomarkers have been presented (Mayer et al., 2018; Mi et al., 2018). Various biomarkers identified in this study have also been validated in such two studies. For instance, in Mayer et al.’s (2018) study, four genes, including NKX2-1, NR2F1, NR2F2, and CITED2, were also identified. The specific biomarkers like NKX2-1 and NR2F2 have been identified in both two previously studies. As discussed above, both genes have been validated by recent publications with solid experimental supports. The identification of these biomarkers that have also been reported in similar studies validated the utility of our method. However, other biomarkers, like Calm1 and Epha5, have not been recognized by previous studies, indicating that our study may discover new biomarkers for different subgroups of CINs, and therefore, provide a new perspective into the subgrouping of cortical interneuron.

### Limitations of this study

As discussed above, we identified a group of functional genes and specific quantitative rules that contribute to the prediction of CIN cell types. However, there remain certain limitations in our analyses. Firstly, the result may be affected by the cell-type specificity of each gene in the dataset we used. For instance, YWHAZ is specifically expressed in projection neurons, but ribosomal protein subunits may be identified in almost each cell. Therefore, YWHAZ is more likely to be identified as a biomarker in a dataset with a lot of projection neurons. Secondly, the interpretation and discussion on the sub-division groups and related biological functions just provided a probable explanation for the machine learning based sub-classification results according to previously reported publications, which may not necessary be the actual biological mechanisms. Thirdly, we only tried to identify CIN cell subgrouping biomarkers in one dataset. Further validation of our results in matched mouse model will be accomplished in the next step of our analyses.

## Conclusion

In this study, machine learning algorithms were applied to predict CIN cell types (CGE, dMGE, and vMGE cells) at the regions of origin by using single-cell transcriptome sequence. Our findings revealed that CIN cell types could be successfully distinguished by their transcriptomic characteristics, especially the CGE cell, suggesting that the diversity of CIN is invoked in the original region before they migrate. Furthermore, a group of genes as well as their combined rules that are important for the prediction of CIN types at the regions of origin were identified and interpreted. Our research may not only provide novel biomarkers for CIN associated myoblasts subtyping but also contribute to deepening the understanding of CIN associated biological processes and related mechanisms.

## Data Availability Statement

Publicly available datasets were analyzed in this study. This data can be found here: https://www.ncbi.nlm.nih.gov/geo/query/acc.cgi?acc=GSE109796.

## Author contributions

TH and Y-DC designed the study. ZL, DW, WG, SZ, LC, and XP performed the experiments. ZL, DW, WG, Y-HZ, and LL analyzed the results. ZL, DW, and WG wrote the manuscript. All authors contributed to the research and reviewed the manuscript.

## Conflict of interest

The authors declare that the research was conducted in the absence of any commercial or financial relationships that could be construed as a potential conflict of interest.

## Publisher’s note

All claims expressed in this article are solely those of the authors and do not necessarily represent those of their affiliated organizations, or those of the publisher, the editors and the reviewers. Any product that may be evaluated in this article, or claim that may be made by its manufacturer, is not guaranteed or endorsed by the publisher.

## Supplementary material

Supplementary Table 1 Feature list generated by mRMR method.

Supplementary Table 2 Performance of RF and RIPPER classifiers with different top features in the list.
